# Quantitative rapid image-based method for coronary artery blood flow and wall shear stress extraction

**DOI:** 10.3389/fbioe.2026.1805161

**Published:** 2026-06-03

**Authors:** Yaofeng Ju, Nelson Tansu, Peter J. Psaltis, Mergen H. Ghayesh

**Affiliations:** 1 School of Electrical and Mechanical Engineering, Adelaide University, Adelaide, SA, Australia; 2 Vascular Research Center, Lifelong Health Theme, South Australian Health and Medical Research Institute (SAHMRI), Adelaide, SA, Australia; 3 College of Health, Adelaide University, Adelaide, SA, Australia; 4 Department of Cardiology, Central Adelaide Local Health Network, Adelaide, SA, Australia

**Keywords:** coronary angiography, image-based biomechanics, pixel intensity convection model, real-time coronary flow assessment, wall shear stress

## Abstract

This paper shows that it is possible to quickly determine the velocity field of coronary blood flow and wall shear stress using a very fast image-based approach on a routine coronary angiogram, which is an alternative approach to reconstructing a target arterial model and performing time-consuming computational fluid dynamics simulations. The method analyzes two frames from a short contrast washout sequence acquired 0.1 s apart and applies a convection-dominant transport model to estimate local projected velocity and instantaneous projected wall shear stress. In four patients with stenoses in the middle third of the left anterior descending coronary artery, the peak wall shear stress within the stenotic segment showed a mean relative difference of 10.3% compared with a same-geometry two-dimensional ANSYS computational fluid dynamics reference. These results suggest that rapid image-only wall shear stress estimation may be used as an adjunctive hemodynamic assessment tool to provide additional information to clinicians when time is critical. However, further studies are still needed for broader validation and for evaluation at multiple time points during the cardiac cycle.

## 1 Introduction

Atherosclerosis, which underlies coronary artery disease, is one of the main causes of morbidity and mortality globally. Both systemic risk factors as well as local hemodynamics play an important role in the development and progression of atherosclerosis. Wall shear stress (WSS) is among the most frequently used descriptors of local hemodynamics. Studies have shown that areas subjected to unusual hemodynamic conditions are associated with the initiation and growth of atherosclerotic plaques and could be involved in the process of lesion remodeling and plaque phenotype. These findings have led researchers and clinicians to map WSS distributions for hemodynamic assessment and for risk stratification. Mapping WSS distributions is usually done through computational fluid dynamics (CFD) on patient-specific models of the target coronary artery segment (Carpenter et al., 2020). Recent coronary CFD studies have also shown that coronary velocity and wall shear stress depend on the assumed flow conditions and rheological model in reconstructed left coronary artery geometries (Chen et al., 2024; Pandey and Yadav, 2022; Rizzini et al., 2022). Unfortunately, this pipeline often involves manual image reconstruction processes as well as extensive computational power requirements, which limits its usability for timely clinical applications.

In this study we presented an efficient two-dimensional (2D) pixel-based image processing technique based on the routine coronary angiogram obtained as a short time series during the contrast agent washout phase. By taking advantage of the intensity of the contrast agent as a marker for the blood flow in each pixel, we were able to calculate the corresponding local velocity vector and then compute the WSS. The intensity of the pixels from the angiograms was considered proportional to the line-integrated iodine contrast along the x-ray beam, as is consistent with the physical principles of x-ray absorption (Hubbell and Seltzer, 1995; Kak and Slaney, 2001). Prior calibration and characterization studies have shown that within clinical ranges, the pixel intensity in the angiograms has a nearly linear response to iodine mass thickness at constant acquisition conditions, which allows us to use image pixel intensity for the calculation of velocity and WSS (Chida et al., 2000; Seibert, 2006). We compared the proposed method with a same-geometry 2D ANSYS CFD reference in four mid left anterior descending (mid-LAD) coronary artery lesions. The peak WSS within the stenotic segment showed a mean relative difference of 10.3% compared with the CFD reference. In its current form, the method is best interpreted as a rapid adjunctive tool for hemodynamic assessment and as a research tool for patient-specific analysis.

## 2 Methods

Coronary angiography cine images were obtained at the Royal Adelaide Hospital (RAH) during routine, clinically indicated procedures. Image processing and analysis were performed at the South Australian Health and Medical Research Institute (SAHMRI) and the University of Adelaide. This study was approved by the Central Adelaide Local Health Network Human Research Ethics Committee (CALHN HREC; approval ref. 14,179).

We have developed an image-driven, 2D approach that provides extracts of blood-flow velocity and WSS based on two frames selected from a short angiographic washout sequence. In this study, the selected frame pairs were taken in early diastole. The two frames used in the analysis were selected during the decreasing-intensity washout phase after contrast injection ceased. In the present method, this short washout interval provides the usable intensity change needed for the calculation of velocity and WSS.

**FIGURE 1:**
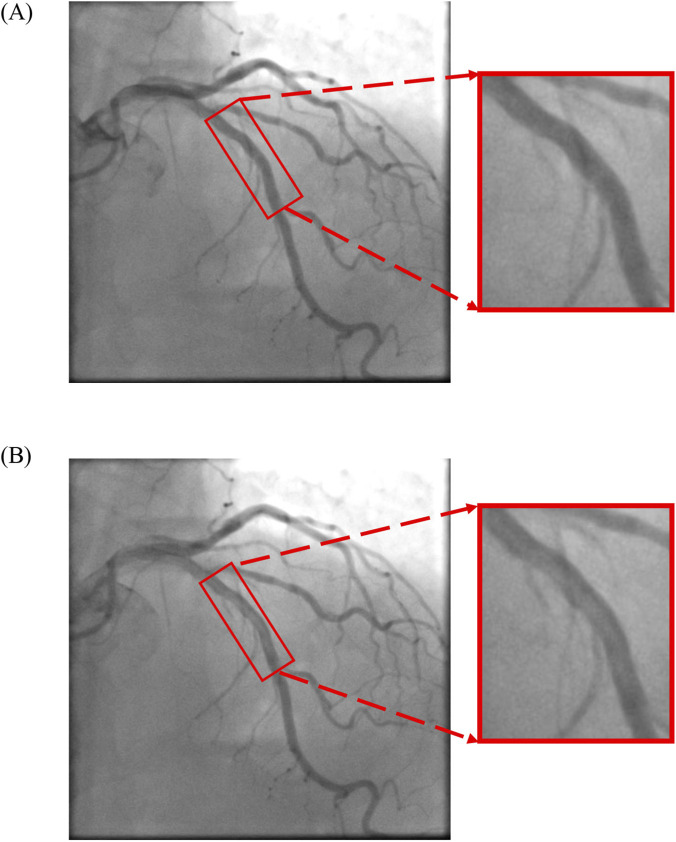
**(A)** Raw angiographic frame acquired immediately after cessation of contrast injection; **(B)** subsequent frame acquired 0.1 s later.

Because the angiographic cine sequence was not ECG gated, the intensity change between two frames may reflect not only contrast washout, but also some residual effect from cardiac motion and small changes in vessel position or shape in the projection image. The selected frames were closely spaced in time to reduce this effect as much as possible. The time interval (Δ*t*) is fixed by the system frame rate at 0.1 s; we therefore use Δ*t* = 0.1 s for each frame pair to operationalize the advection-dominant assumption (see Figure 1 for raw frames at *t*_1_ and *t*_2_ = *t*_1_ + Δ*t*). In principle, the present method could be extended to multi-frame analysis if usable contrast variation were available over more frames. However, in the present study the usable washout signal was only available over a short interval of a few frames. After that, the contrast became too diluted, and the intensity change was no longer sufficient for reliable velocity and WSS calculation. For this reason, the current method is limited to a selected washout phase and cannot yet provide cycle resolved WSS over the full cardiac cycle.

**FIGURE 2:**
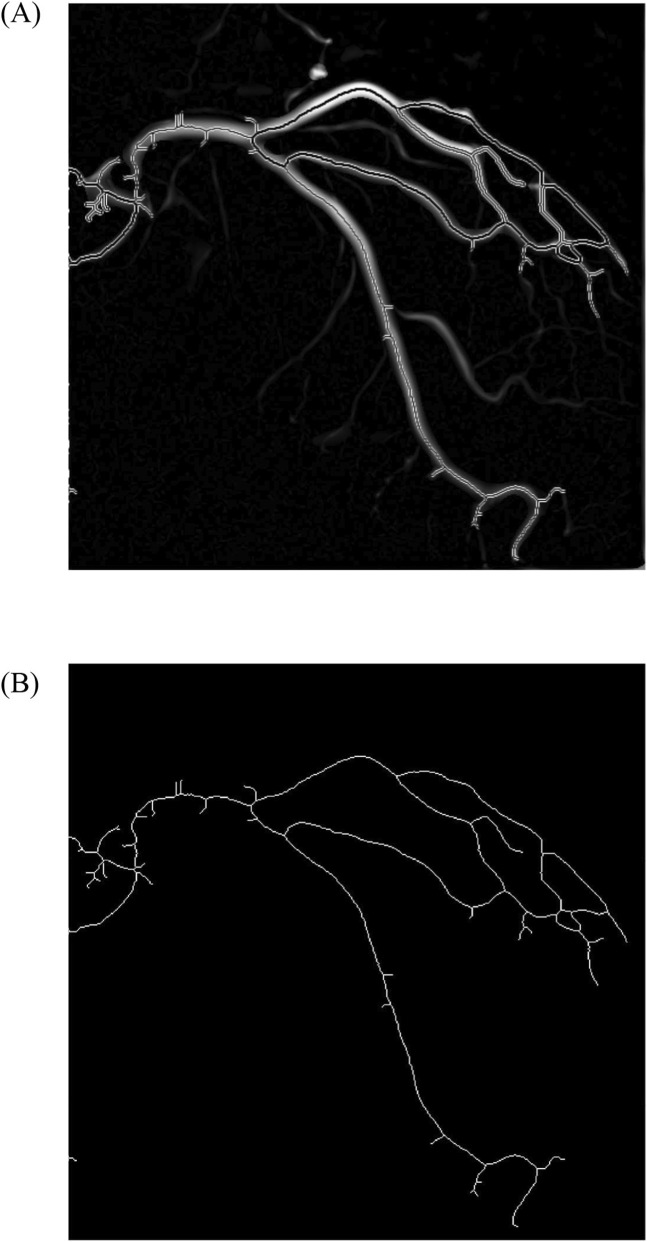
**(A)** Frangi-filtered view with the single-pixel skeleton overlaid; **(B)** the corresponding single-pixel skeleton.

All images were converted to grayscale and resized to 512 × 512 pixels. Preprocessing then applied a Gaussian filter using a 3 × 3 kernel with *σ* = 2 pixels. Next, vessel enhancement was performed using a multi-scale Frangi filter with scales ranging from 1.0 to 10.0 pixels. These preprocessing settings were kept fixed across the analyzed cases. The resulting images are then morphologically thinned to produce a single-pixel-wide skeleton (see Figure 2). In the downstream calculation, the fixed frame interval was Δ*t* = 0.1 s, a small lower bound was imposed on the gradient magnitude before inversion, and a fixed clipping threshold was used to remove obvious velocity outliers.

A Gaussian filter can be expressed using a 2D kernel function $G\left(x,y\right)$ , which can be written as Equation 1 (Haddad and Akansu, 1991):

$$G\left(x,y\right)=\frac{1}{2\pi \sigma^{2}}e^{\left(‐\;\frac{x^{2}+y^{2}}{2\sigma^{2}}\right)}$$ (1)

When implementing the filtering operation, for any pixel $\left(u,v\right)$ in the original image I , the Gaussian-smoothed image $I_{gauss}$ can be computed using Equation 2:

$$I_{gauss}\left(u,v\right)=\sum_{x=‐r}^{r}\sum_{y=‐r}^{r}I\left(u‐x,v‐y\right)\;G\left(x,y\right)$$ (2)

indicating that, at each pixel, a weighted summation is performed over a local neighborhood to achieve a smoothing effect.

Upon completion of the Gaussian filtering, an image with relatively reduced noise is obtained. In the case of particularly thin vascular structures, σ must be carefully controlled to prevent excessive smoothing that might lead to the loss of fine vessel information. In the present implementation, a 3 × 3 Gaussian kernel with σ = 2 pixels was used for all analyzed cases. The Gaussian smoothing step was used to reduce high-frequency cine noise before vessel enhancement. A smaller σ would retain more local detail but increase noise sensitivity, whereas a larger σ would increase smoothing at the expense of vessel-edge sharpness.

Next, Frangi filtering (Frangi et al., 1998) is carried out. The core principle of Frangi filtering is to detect tubular structures using a multi-scale Hessian matrix (Frangi et al., 1998). For each pixel $\left(u,v\right)$ in the image, a set of scales $\sigma_{1},\sigma_{2},\ldots ,\sigma_{n}$ is predefined. At each scale, the second-order partial derivatives, including $D_{xx}$ , $D_{xy}$ , $D_{yy}$ are computed.

At each scale σ , the second-order partial derivatives of the image can be obtained by convolving the image with the second derivatives of a Gaussian kernel, as shown in Equation 3:

$$D_{xx}\left(x,y;\sigma \right)=\frac{\partial^{2}}{\partial x^{2}}\left(I_{gauss}\left(x,y;\sigma \right)\right)$$ (3)

where can be viewed as the smoothed version of the original image at scale. In the present implementation, vessel enhancement was performed using a multi-scale Frangi filter with scales ranging from 1.0 to 10.0 pixels, and the same preprocessing settings were kept across the analyzed cases.

**FIGURE 3:**
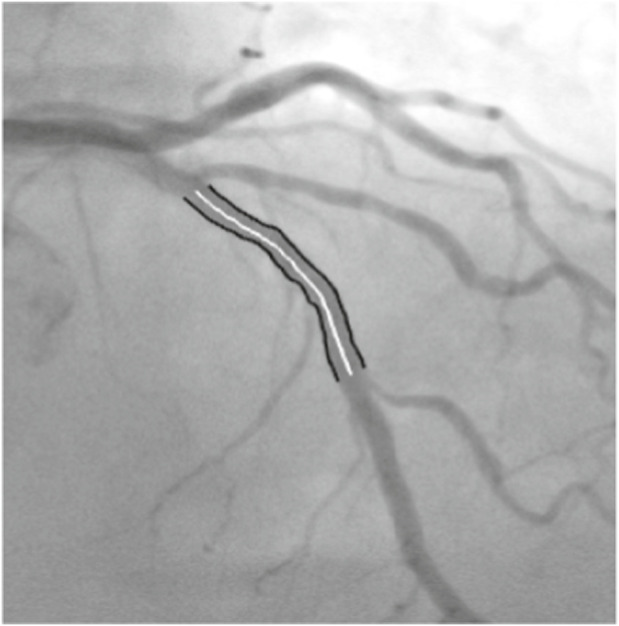
Centreline extraction and artery wall segmentation overlaid on the angiogram; the centreline is shown in white and the wall contours in black.

The use of a skeletonized image as a basis for the extraction of vascular structures begins with the manual definition of proximal and distal endpoints for the vessel segment of interest. These endpoints are used for constraining a Dijkstra-based minimum-cost path search on an 8-connected pixel graph (Dijkstra, 2022) with edge weights defined by a composite cost map derived from image edge and gradient information. The result of this process is a discrete arterial centerline, which is subsequently modelled using cubic B-spline interpolation to generate a smooth centerline (white curve in Figure 3).

The arterial wall segmentation is delineated using an interactive, graph-based live-wire method on the same graph. The user selects a series of seed points, and the software determines the minimum-cost path between successive seeds; path costs are determined from the composite cost map that uses edge and gradient information to reduce clutter from the image background while emphasizing vessel margins (Barrett and Mortensen, 1997; Mortensen and Barrett, 1998; Jie and Ning, 2012; Li et al., 2017; Chodorowski et al., 2005). Further, each of the extracted path segments is smoothed using a B-spline and may be sub-divided to provide a dense wall coordinate set (black curves in Figure 3).

**FIGURE 4:**
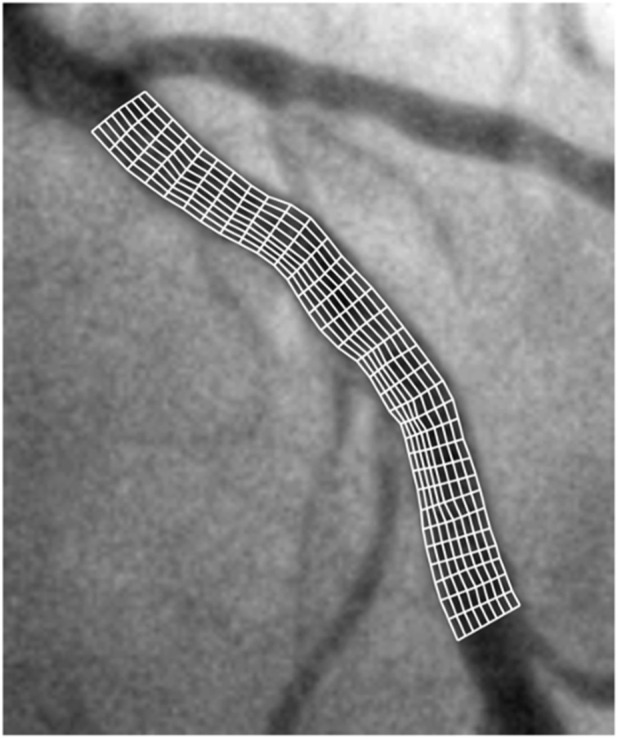
Surface mesh extracted from the LAD segment on the coronary angiogram.

With these geometric elements, we create a set of cross-sections spanning from the left artery wall, through the center line, to the right artery wall for each sampling location. We subdivide each of the cross-sections into many parts to produce a dense 2D grid of points illustrated in Figure 4. Using the pixel coordinates (X) associated with a given mesh node, we are able to obtain intensity values consistently from both of the angiographic frames. By normalizing the intensity values obtained from the two frames, we are able to determine an approximation of the contrast concentration C1 and C2 at times t1 and t2, respectively (Slim et al., 2013). The local contrast changes over time can be expressed as Equation 4:

$$\frac{dC}{dt}=\frac{C_{2}\left(i,j\right)‐C_{1}\left(i,j\right)}{\Delta t}$$ (4)

In the selected washout interval, the contrast intensity generally decreased with time, so ∂ *C*/ ∂ *t* was negative. Simultaneously, we compute the spatial gradient of *C*_1_​ (the earlier time point) using the Scharr operator, which has improved rotational symmetry compared with operators such as Sobel and can better capture subtle vessel edges (Li et al., 2017).

Under a simplified advection-dominant model, we calculate the local flow velocity from the advection equation shown in Equation 5 (Ching et al., 2017; Batchelor, 2000)

$$\frac{\partial C}{\partial t}+v\left(i,j\right)\;\cdot \nabla C=0$$ (5)

assuming that contrast transport is dominated by convection and that diffusion is neglected during the short washout interval (De Paoli et al., 2020). Because the present method uses only a single angiographic projection, only the projected in-plane motion can be represented directly. Out-of-plane motion is not recovered separately, and the image intensity is interpreted as projected contrast information along the x-ray beam path. In the present implementation, the frame-to-frame intensity change was then used to determine the local speed magnitude, and the corresponding projected velocity vector was represented along the local vessel centerline direction. The gradient magnitude was regularized by imposing a small lower bound 1 × 10^−6^ before inversion so that division by near-zero values did not destabilize the calculation. Vectors above a fixed clipping threshold of 1.0 in the internal image-based velocity units were discarded as outliers.

To compute WSS, we use near-wall velocity gradients. The near-wall distance used for WSS calculation was determined locally from the distance between adjacent mesh nodes in the normal direction near the vessel wall. In the present study, this locally defined near-wall distance was used as a simple and stable estimate for the proof-of-concept calculation. It kept the calculation close to the wall while reducing sensitivity to direct differentiation exactly at the segmented boundary. The present implementation used a local finite-difference estimate rather than a multi-point wall-normal fit. Treating blood as a Newtonian fluid with viscosity μ=0.0035 Pa·s , we calculate WSS according to Equation 6 (Samyn and La Disa, 2016; Benard et al., 2006).

$$WSS=\mu \cdot \frac{\partial u_{t}}{\partial n}$$ (6)

The computed values are mapped to the extracted vessel walls to generate a color-coded WSS visualization. WSS is displayed over the angiogram to support clinical inspection of regions associated with elevated hemodynamic risk.

## 3 Results

Four clinical cases with stenosis in the mid-LAD coronary arteries were used to evaluate the proposed method. The two analyzed cine frames were acquired 0.1 s apart during the decreasing-intensity washout phase after contrast injection ceased. After the Gaussian smoothing, vessel enhancement, and centerline/wall extraction, we generated a uniformly sampled grid of cross-sectional points for the analyzed mid-LAD segment.

### 3.1 Results based on the proposed method

By comparing pixel intensity and spatial gradients between the two frames, we calculated a 2D velocity field and observed higher flow speeds within the narrowed region of the flow path. These velocity patterns are as predicted by the fundamental principles of fluid dynamics as well as those of hemodynamics where a narrowing will accelerate flow locally.

**FIGURE 5:**
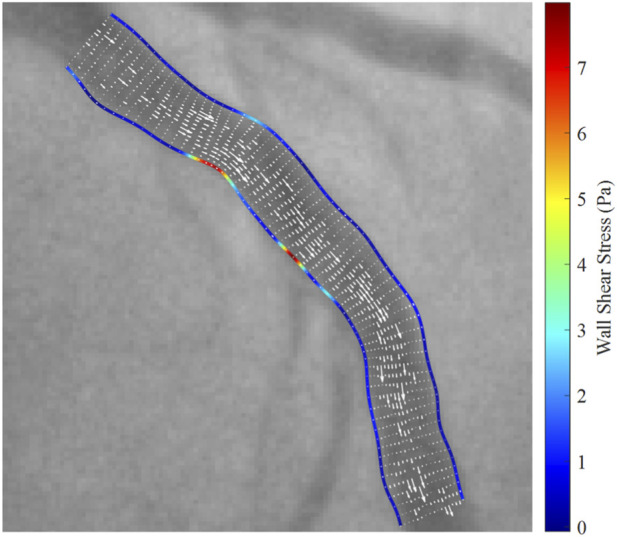
Wall shear stress distribution overlaid with velocity vectors obtained by using the current method.

In addition, using the computed near wall velocity gradient we were able to determine the WSS distribution and visualized it as a color map (see Figure 5). The peak WSS value at the site of the stenosis was measured to be 8.14 Pa and therefore represents an area of high WSS values that may contribute to the development and/or instability of plaques.

To provide a basic physical-consistency check, we also compared the estimated mass flow rates at the proximal and distal ends of the stenotic segment under a circular cross-sectional assumption. In this case, the proximal-to-distal mass flow rate mismatch was 8.8%, indicating reasonable internal consistency of the derived velocity field across the analyzed stenotic span. Under the same assumption, the estimated mass flow rate was on the order of 24–26 mL/min. This analysis was used only as an internal consistency check within the present 2D framework and was not interpreted as a direct measurement of the true three-dimensional coronary flow rate.

### 3.2 Validation of the results with those of CFD

**FIGURE 6:**
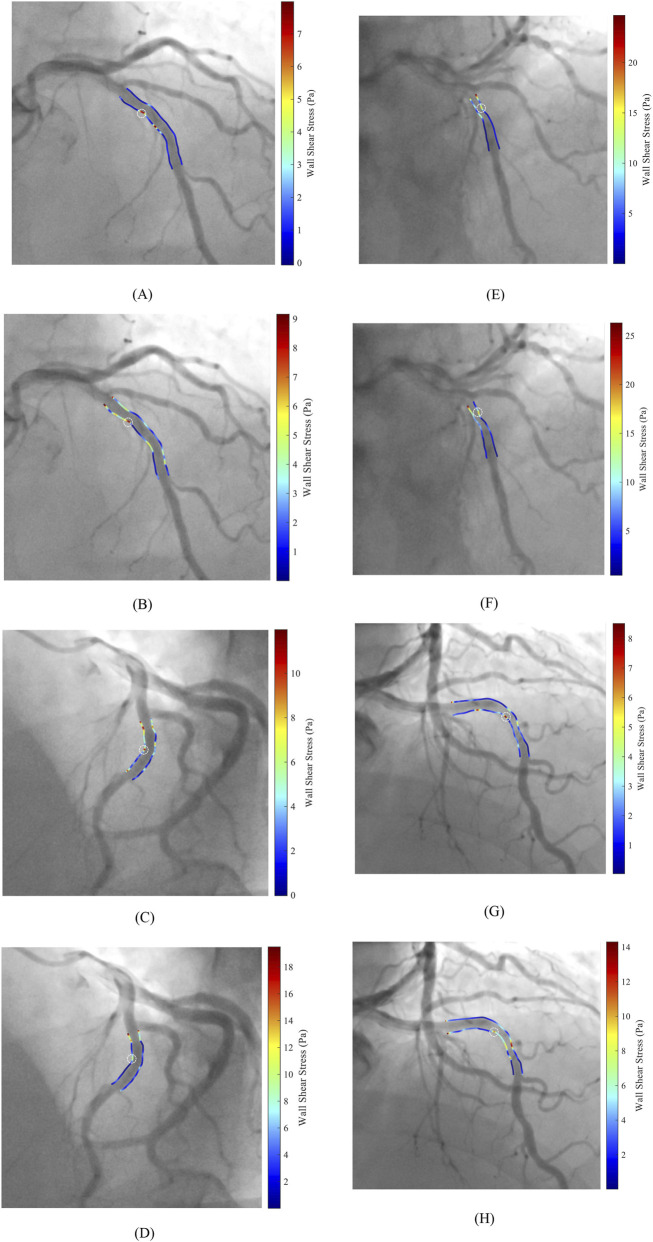
Wall shear stress overlays co-registered to angiogram frames with stenoses circled by white dashed lines. **(A)** Case 1: current method; **(B)** Case 1: ANSYS 2D CFD; **(C)** Case 2: current method; **(D)** Case 2: ANSYS 2D CFD; **(E)** Case 3: current method; **(F)** Case 3: ANSYS 2D CFD; **(G)** Case 4: current method; **(H)** Case 4: ANSYS 2D CFD.

In four mid-LAD cases, image-based WSS was obtained on the angiograms and rendered as continuous wall-attached distributions (see Figure 6). For validation, the same segmented 2D lumen extracted from the analyzed angiographic projection was used in ANSYS Workbench (Version 2022 R2, ANSYS, Inc., Canonsburg, PA) to construct a same-geometry 2D CFD reference. No 3D reconstruction was introduced in this comparison. In this reference model, blood was treated as a Newtonian fluid with a viscosity of 0.0035 Pa·s and a density of 1,060 kg/*m* ^3^, and the wall was treated as rigid. A constant inlet velocity of 0.3 m/s and a constant outlet pressure of 13,300 Pa were applied (Gholipour et al., 2018; Kuchumov et al., 2021). The corresponding Reynolds numbers were 302.2, 203.1, 146.4, and 342.2 for Cases 1–4, respectively, indicating laminar flow in the 2D ANSYS reference model. The 2D ANSYS reference model was meshed using a quadrilateral-dominant method with free face meshing of Quad/Tri type and an element size of 0.1 mm. The transient simulation used a time step of 0.005 s for 200 time steps, with 60 iterations per time step. The ANSYS simulation was run as a transient model with a constant inlet velocity, and the simulation time was chosen long enough for the solution to reach an effectively steady state.

**FIGURE 7:**
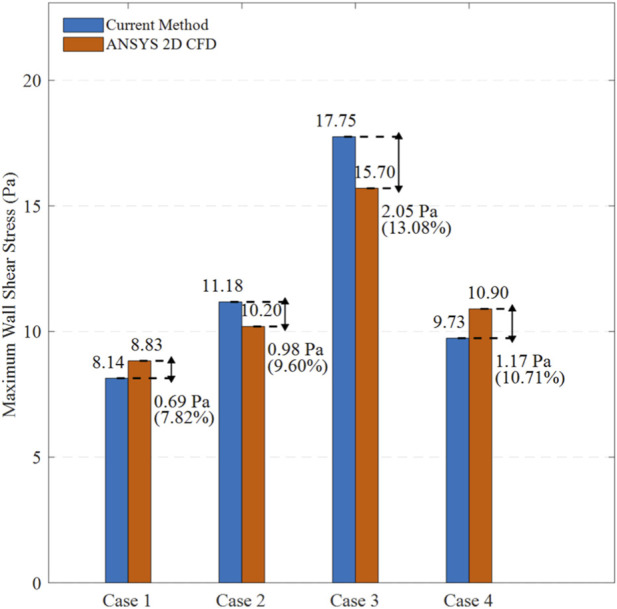
Peak wall shear stress in the stenotic region: comparison of the current image-based method with ANSYS 2D CFD.

**FIGURE 8:**
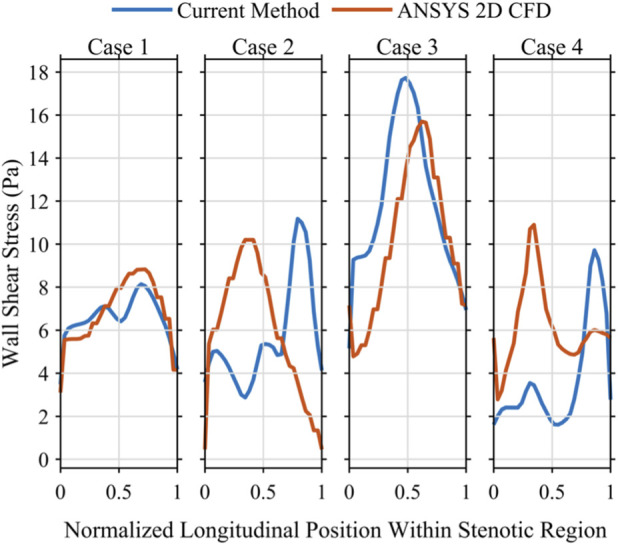
Wall shear stress versus normalized longitudinal position within the stenotic region: comparison between the Current Method and ANSYS 2D CFD.

For each case, the peak WSS was taken as the maximum WSS within the predefined stenotic subsegment rather than the absolute maximum over the entire analyzed vessel (see Figure 7). This definition was used consistently for both the image-based results and the same-geometry 2D ANSYS reference. Before peak extraction, the WSS curves were smoothed along the vessel using a cubic smoothing spline with a smoothing parameter of 0.9. Therefore, the reported peak WSS was obtained from the smoothed wall-attached distribution within the stenotic subsegment rather than from a single raw node. The mean absolute difference was 1.22 Pa (median 1.07 Pa; range 0.69–2.05 Pa), and the mean relative difference was 10.3% (median 10.2%; range 7.8%–13.1%). To further examine the spatial agreement between the two methods, WSS was plotted against normalized longitudinal position within the predefined stenotic region for all four cases (see Figure 8). These line plots showed that the current image-based method reproduced the main WSS trend and identified the principal high-WSS region within the analyzed segment, although some local differences in profile shape and peak position remained between the current method and the same geometry 2D ANSYS reference. Accordingly, the present comparison should be interpreted as spatial proof-of-concept agreement rather than exact pointwise equivalence.

## 4 Discussion

The primary advantage of this new methodology is an image-only process that provides a rapid alternative to conventional 2D CFD workflows for evaluating coronary hemodynamics, using post-injection contrast washout in routine angiograms to calculate velocity fields and WSS. The methodology eliminates the need for extensive arterial modelling and is therefore compatible with clinical demands for timely, data-driven hemodynamic information. In its current form, the method is best interpreted as a rapid adjunctive tool for hemodynamic assessment in the catheterization laboratory and as a research tool for patient-specific analysis. It is not intended to replace pressure-derived indices such as FFR or iFR. Because the present method can rapidly extract WSS information directly from routine angiographic images, it may provide additional hemodynamic information in the stenotic region when time is limited. At the current stage, this information should be interpreted as complementary rather than standalone. In the present workflow, after two adjacent angiographic frames from the washout phase were selected, the user manually specified the start and end points of the vessel segment of interest. The remaining steps were then carried out automatically by the software. In the current MATLAB research prototype, the automated computation required approximately 5 s on a MacBook Pro with an M3 Max chip. The manual interaction time may vary depending on operator experience.

In the present study, only two frames from a short washout interval were used. Therefore, the method provides projected instantaneous WSS at the selected phase rather than cycle-averaged indices such as time-averaged WSS or oscillatory shear index. For this reason, peak WSS in the predefined stenotic subsegment was used as the main comparison metric in this proof-of-concept study. This quantity reflects the local high-shear region associated with flow acceleration in the stenotic segment. The present method provides projected instantaneous WSS at the selected washout phase. Therefore, it should not be interpreted as a direct substitute for long-term hemodynamic markers such as low time-averaged WSS or oscillatory shear index. In the present framework, high WSS is the most direct hemodynamic feature that can be obtained from the current two-frame washout method. In the current study, the highest WSS values were found in the stenotic region, so this was used as the main quantity for comparison.

A second advantage of the proposed methodology is that its accuracy is expected to improve with advances in imaging technology. Higher spatial resolution reduces partial-volume effects at vessel boundaries and increases the spatial intensity gradient, whereas higher cine frame rates provide finer temporal sampling of contrast transport during washout, thereby reducing temporal discretization error in the calculated velocity field. Since the methodology is explicitly physics-based and contains no trainable parameters, improvements in detector resolution and cine rate can translate directly into improved fidelity of the calculated WSS, without the need for retraining or generation of CFD-derived training datasets, unlike many deep-learning-based surrogate methodologies (Gharleghi et al., 2022; Su et al., 2020; Alamir et al., 2024). Thus, the methodology can benefit from advances in angiography technology while retaining a direct mechanistic connection between image data and the derived hemodynamic quantities.

The study has a limitation due to its reliance on local image intensity as a proxy for contrast concentration. As a result, the methodology is inherently sensitive to vessel overlap and foreshortening. Because the present method uses only a single angiographic projection, only the projected in-plane motion can be represented directly. Out-of-plane motion is not recovered separately, and the image intensity is interpreted as projected contrast information along the x-ray beam path. To date, we limit the analysis to non-overlapping mid-LAD segments with limited foreshortening. The present formulation does not recover a full three-dimensional cross-sectional geometry from a single projection. In the present implementation, velocity outlier rejection affected only a small fraction of the calculated vectors. In Case 1, 14 of 1,105 candidate velocity vectors were removed using the fixed velocity threshold, corresponding to a rejected fraction of 1.27%, whereas 98.73% of the calculated velocity vectors were retained. The removed outliers were mainly located away from the vessel wall. Because the present study focused on WSS estimation, the near-wall part of the velocity field was the main quantity of interest. The calculated velocity field in the present study should be interpreted as a projected velocity representation from a single angiographic view. The main purpose of the current formulation was the estimation of WSS rather than full validation of the interior velocity field. Because the present method is based on a single projection, the interior velocity field is more affected by projection effects and should not be interpreted in the same way as a full three-dimensional CFD solution. In the present study, the main quantity of interest was the near-wall velocity information used for WSS estimation. External flow rate validation was not included in the present study. This will be more appropriate in a future study designed specifically for broader flow analysis. Although the peak WSS magnitude was captured reasonably well, the line-plot comparison indicated that accurate spatial localization of the peak remained challenging in certain geometries, particularly in Cases 2 and 4, where the peak position differed from the same geometry 2D ANSYS reference. Some of these limitations may be reduced by integrating coronary segmentation methodologies developed for invasive coronary angiography. A recent study (Zhao et al., 2021) described an automated framework in which a deep learning model segmented coronary arteries in invasive angiograms and subsequent processing extracted vascular centerlines, computed local diameters, and quantified stenosis severity. A similar segmentation-and-centerline strategy, in conjunction with multi-view angiography when available, may allow identification of individual branches, localization of overlapping segments, and provision of complementary geometric information for image-based WSS determination.

To the authors’ knowledge, we are not aware of prior methods that determine quantitative coronary WSS directly from routine 2D angiographic image sequences using only pixel intensities and frame-to-frame intensity changes, without requiring 3D reconstruction, CFD pre-computation, or training data. This positions the present method differently from angiography-based 3D reconstruction followed by CFD, from angiographic velocimetry methods that estimate flow or velocity but do not directly provide WSS, and from deep-learning-based WSS surrogates that require training on CFD-derived datasets. The proposed methodology provides an image-only, physics-based route to quantitative WSS that naturally interfaces with standard angiographic workflows and can be implemented with limited computational resources. In its current form, the method is best interpreted as an adjunctive, single-projection tool rather than a standalone clinical decision metric.

## 5 Conclusion

In this study, we presented a quantitative image-based method to determine coronary blood-flow velocity and wall shear stress (WSS) from standard angiographic frames. The main findings and implications of this work can be summarized as follows:

- A self-contained framework was developed to derive coronary blood-flow velocity and WSS directly from routine angiographic image sequences.
- The method integrates vessel segmentation, normal-based wall detection, and a simplified advection model to describe frame-to-frame contrast intensity changes.
- This framework enables quantitative extraction of hemodynamic information from standard angiography without requiring complex additional imaging or extensive manual intervention.
- The low user input requirement and short computation time indicate that the method is suitable for rapid analysis and may be adaptable for near-real-time assessment.
- The proposed approach may provide a practical supplementary tool for hemodynamic evaluation in interventional cardiology and may assist the interpretation of coronary artery disease.
- Further validation in larger datasets and broader clinical settings is still needed before routine clinical application.

## Data Availability

The original contributions presented in the study are included in the article/Supplementary Material, further inquiries can be directed to the corresponding authors.
