# Supplementary material for: Quantitative rapid image-based method for coronary artery blood flow and wall shear stress extraction
Source: Front Bioeng Biotechnol. 2026 Jun 3;14:1805161. doi: 10.3389/fbioe.2026.1805161 (PMC13272375; doi:10.3389/fbioe.2026.1805161)
Supplement: Supplementary file 1 [file Supplementaryfile1.docx]

Supplementary Material

## Supplementary Figures


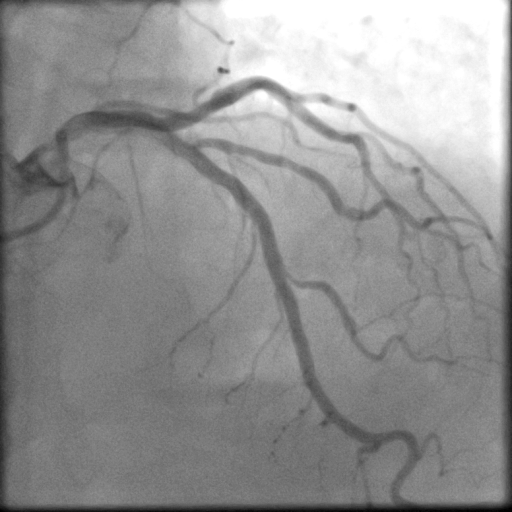


(A)


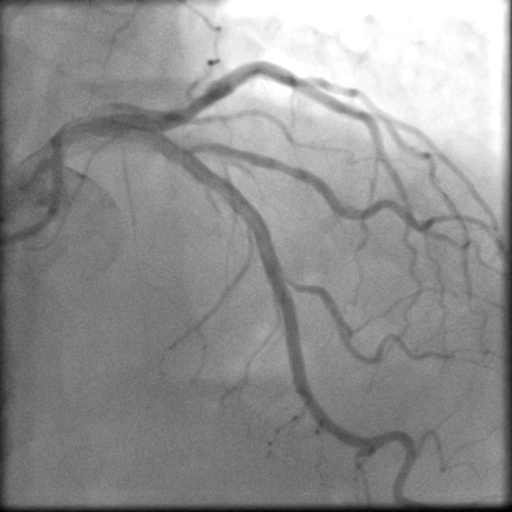


(B)

**Figure 1.** (A) Raw angiographic frame acquired immediately after cessation of contrast injection; (B) subsequent frame acquired 0.1 s later.


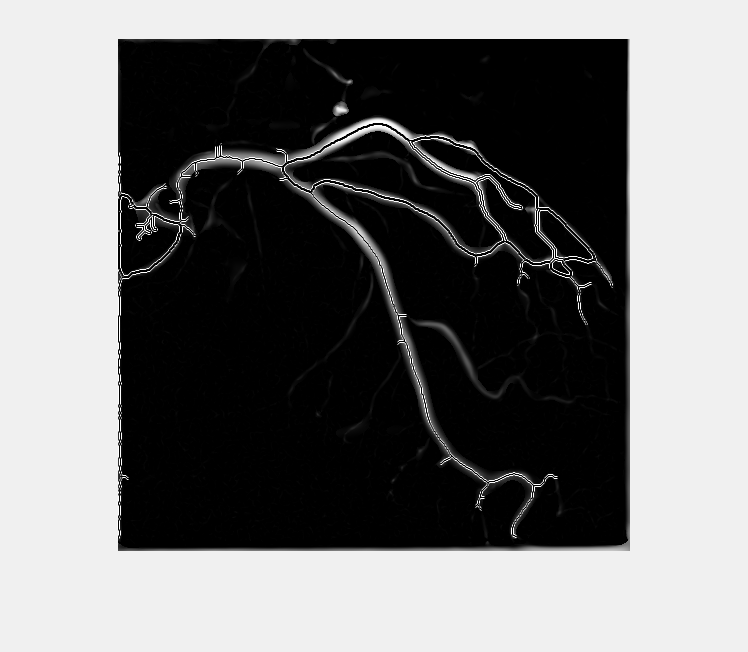


(A)


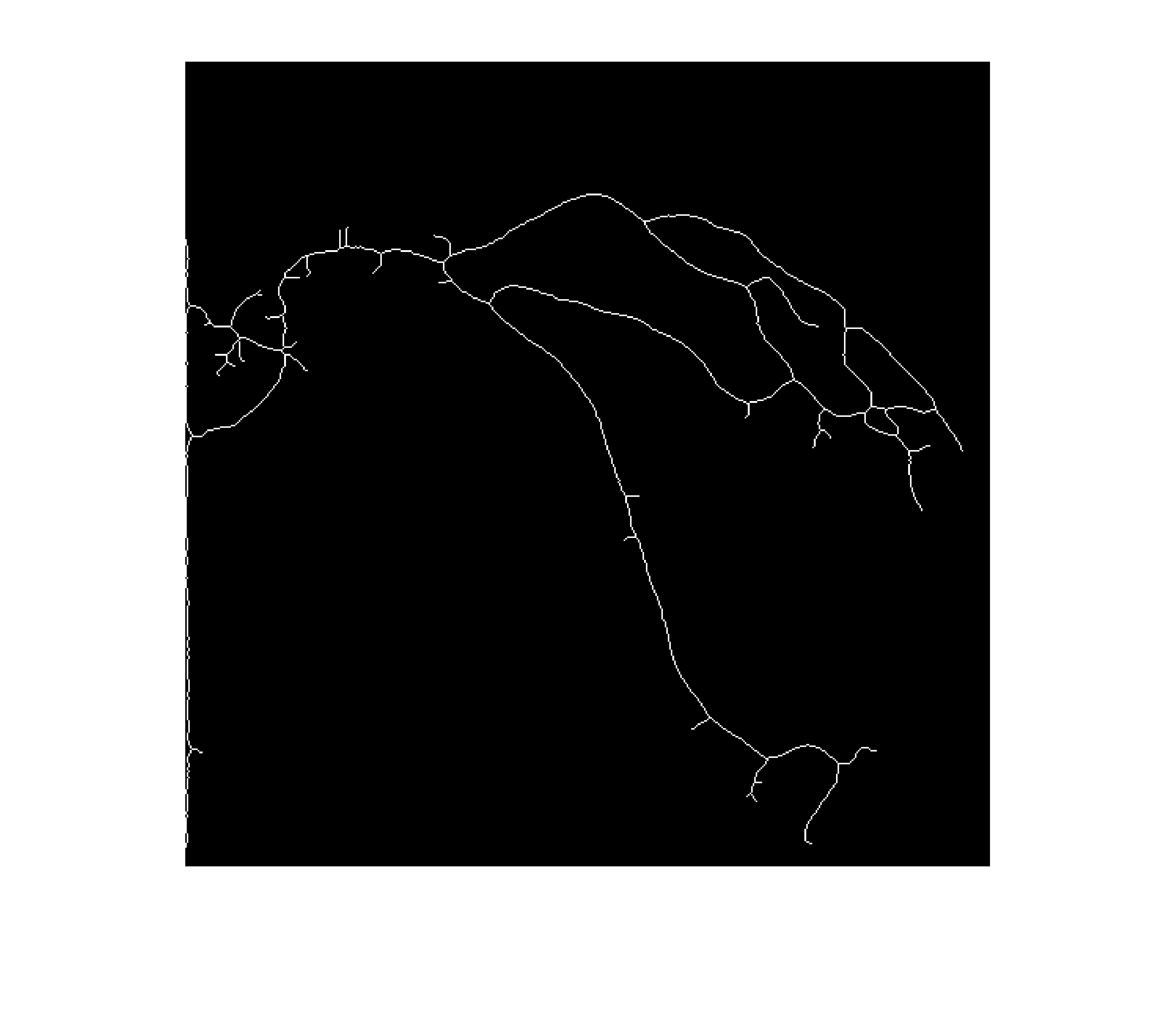


(B)

**Figure 2.** (A) Frangi-filtered view with the single-pixel skeleton overlaid; (B) the corresponding single-pixel skeleton.


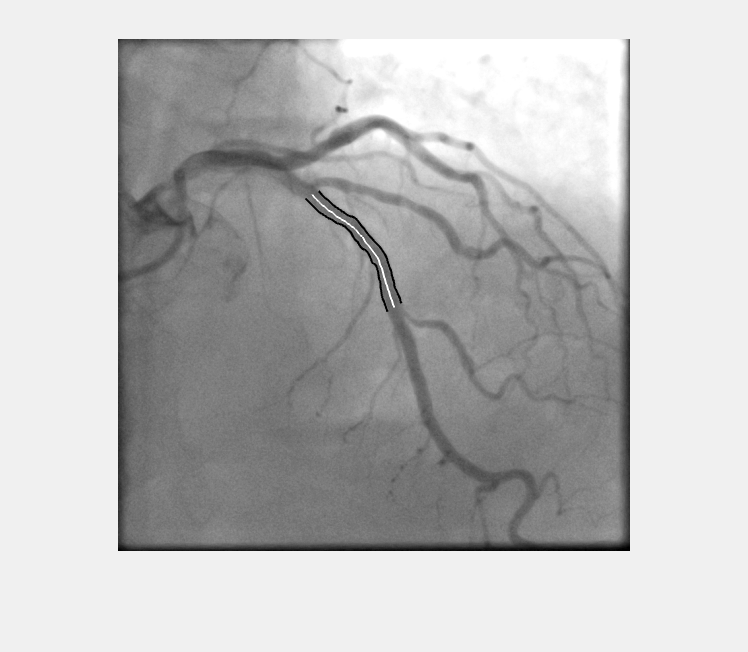


**Figure 3.** Centreline extraction and artery wall segmentation overlaid on the angiogram; the centreline is shown in white and the wall contours in black.


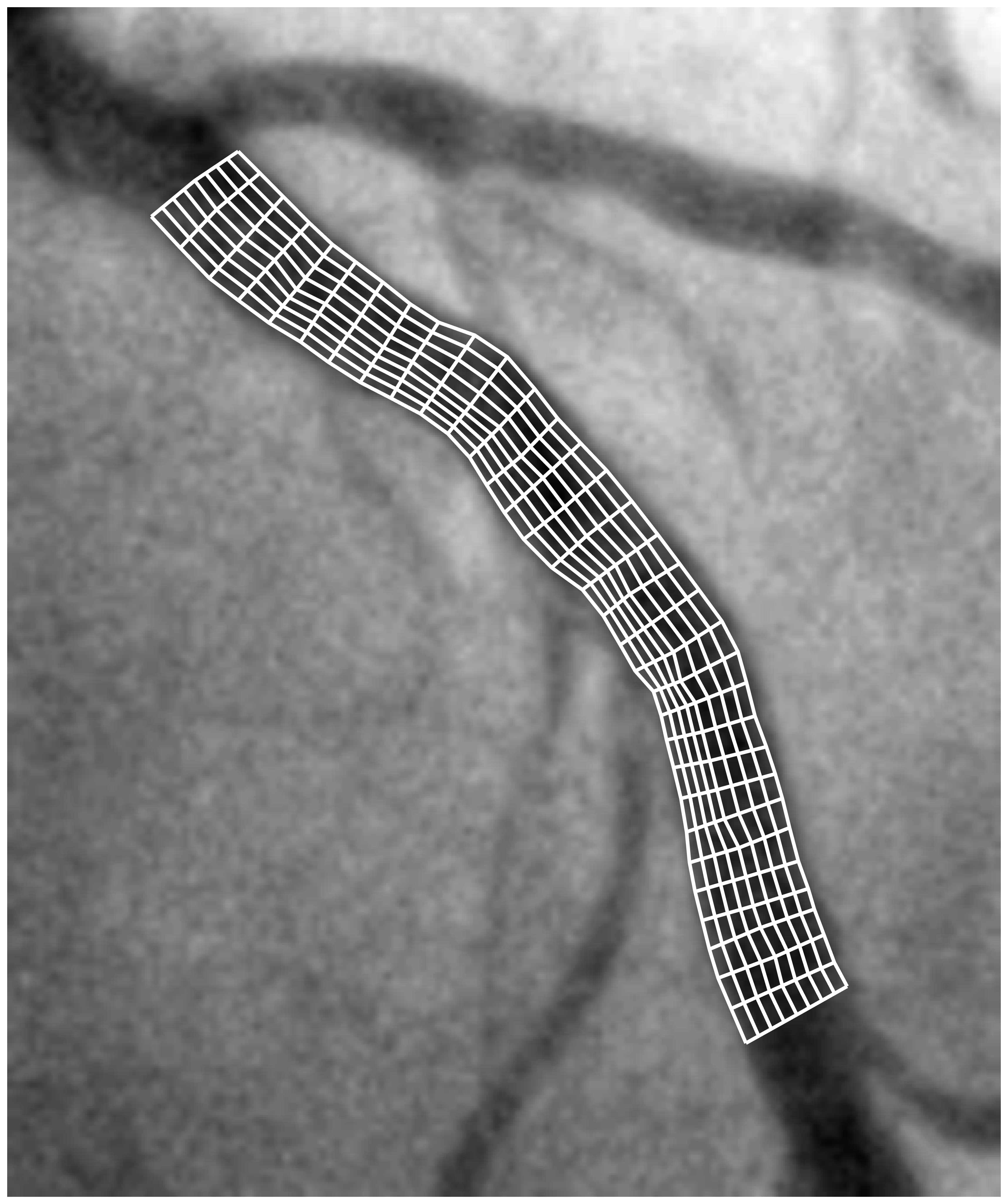


**Figure 4.** Surface mesh extracted from the LAD segment on the coronary angiogram.

**Figure 5.**  Wall shear stress distribution overlaid with velocity vectors obtained by using the current method.

(A)

(B)

(C)

(D)

(E)

(F)

(G)

(H)

**Figure 6.** Wall shear stress overlays co-registered to angiogram frames with stenoses circled by white dashed lines. (A) Case 1: current method; (B) Case 1: ANSYS 2D CFD; (C) Case 2: current method; (D) Case 2: ANSYS 2D CFD; (E) Case 3: current method; (F) Case 3: ANSYS 2D CFD; (G) Case 4: current method; (H) Case 4: ANSYS 2D CFD.

**Figure 7.** Peak wall shear stress in the stenotic region: comparison of the current image-based method with ANSYS 2D CFD.
